# Comparative Performance of a New SARS-CoV-2 Rapid Detection System

**DOI:** 10.1128/Spectrum.00205-21

**Published:** 2021-10-13

**Authors:** Gianni Gori Savellini, Gabriele Anichini, Chiara Terrosi, Shibily Prathyumnan, Claudia Gandolfo, Stefano Marini, Maria Grazia Cusi

**Affiliations:** a Department of Medical Biotechnologies, University of Siena, Siena, Italy; b Microbiology and Virology Unit, S. Maria alle Scotte University Hospital, Siena, Italy; c Dati & Ricerca S.r.l., Rome, Italy; Quest Diagnostics Nicols Institute

**Keywords:** SARS-CoV-2, molecular diagnosis, diagnostic platform

## Abstract

The extraordinary global demand for reagents and diagnostic instruments needed for timely detection of severe acute respiratory syndrome coronavirus 2 (SARS-CoV-2) infection has rapidly affected their availability. In order to meet diagnostic needs, it has been necessary to develop new diagnostic procedures. To date, molecular diagnostic tools have represented the gold standard for diagnosis of SARS-CoV-2 infection, and thus an alternative and real-time PCR system was required. To this aim, a molecular rapid test which works with direct real-time RT-PCR may be a relevant aid. In the present work, the accuracy, sensitivity, and specificity of the bKIT Virus Finder COVID-19 rapid molecular test by Hyris Ltd. was evaluated. Moreover, the influence of a different swab storage medium composition was examined relative to that of a routinely used comparator assay. The Hyris Ltd. assay showed an overall agreement of 100% with the comparator based on a panel consisting of 74 retrospective positive nasopharyngeal swabs (NPSs), collected either in universal transport medium (UTM) or using ESwab. No false-positive result was achieved on samples that previously tested negative. Cross-reactivity screening on microorganisms that commonly colonize the human upper respiratory tract was not detected, excluding the risk of false-positive results. Simultaneously, drugs frequently administered to cure respiratory diseases did not interfere with the analytical performance of the assay. Our results showed that the Hyris Ltd. bKIT Virus Finder COVID-19 is a reliable assay for rapid qualitative detection of SARS-CoV-2, providing the advantage of less complex and unambiguous interpretation of results. Indeed, skilled technicians are not required, and thus the Hyris system is suitable as a rapid and easy system for SARS-CoV-2 diagnosis.

**IMPORTANCE** In order to overcome the increased demand for diagnostic tools for the timely detection of SARS-CoV-2 infection, we tested the bKIT Virus Finder COVID-19 molecular rapid test by Hyris Ltd. The new system was confirmed as a reliable assay for rapid SARS-CoV-2 detection, since sensitivity and specificity parameters were fully satisfied. Moreover, the bKIT Virus Finder COVID-19 provides the advantage of easy results interpretation, since skilled technicians are not required, and thus the Hyris system is a valuable SARS-CoV-2 rapid diagnosis system.

## INTRODUCTION

Severe acute respiratory syndrome coronavirus 2 (SARS-CoV-2), responsible for coronavirus disease 2019 (COVID-19), was first reported in 2019 in Wuhan, China, and the World Health Organization subsequently declared it a pandemic (https://www.who.int/emergencies/diseases/novel-coronavirus-2019). The high incidence of virus diffusion and COVID-19 reported during the first outbreak overwhelmed the capability of health care systems, since a rapid diagnostic procedure was not yet present. Moreover, also during the second outbreak, a simple, sensitive, and rapid diagnostic tool represented an important challenge to reduce the risk of SARS-CoV-2 transmission (1, 2). Therefore, the rapid identification of SARS-CoV-2-positive patients still represents a critical aspect in COVID-19 management and is highly required for efficient and timely isolation of patients. The quantitative reverse transcription-PCR (RT-qPCR) assay for SARS-CoV-2 on nasopharyngeal swabs (NPSs) or bronchoalveolar lavage fluid (BAL) samples represents the gold standard procedure for COVID-19 diagnosis (3–5). Several diagnostic strategies have been quickly developed, including fully automated RT-qPCR, encompassing RNA extraction and direct report of results, and RNA extraction-free RT-qPCR systems (6, 7). High test sensitivity and short time to results are mandatory for SARS-CoV-2 diagnosis. Indeed, some nucleic acid amplification tests (NAATs) allow single samples to be run on demand, providing results in less than 1 h with no need for highly skilled laboratory technicians (8, 9). In the effort to develop an alternative diagnostic method, Hyris Ltd. is offering an innovative and comprehensive product for COVID-19 testing in the form of the Hyris system implemented with a new diagnostic assay for SARS-CoV-2. In this study, we examined the sensitivity and specificity of the direct real-time RT-PCR method (without RNA extraction) of the bKIT Virus Finder COVID-19. Furthermore, we assessed the influence of swab transport medium on rRT-PCR performance, demonstrating that the limit of viral detection is highly affected by the use of saline solution storage medium compared to universal transport medium (UTM), as previously reported by other competitors (7). We showed that Hyris bKIT Virus Finder COVID-19 assays provide an efficiency similar to that of the comparator without risks of cross-reaction effects or false-negative results, supporting sensitivity parameters described during preliminary studies (10, 11). Importantly, the simplicity and diagnostic quality standards of the bKIT Virus Finder COVID-19 make the Hyris system ideal for a SARS-CoV-2 rapid diagnosis.

## RESULTS

### Sensitivity performance of bKIT Virus Finder COVID-19.

The sensitivity of the bKIT Virus Finder COVID-19 detection kit was evaluated at an early step by determining the limit of detection (LoD) on both live SARS-CoV-2 virus and a defined number of viral genome copies. Live virus was diluted in pools of previously tested negative NPS and BAL matrices in order to obtain a final virus titer ranging from 1 × 10^4^ 50% tissue culture infective doses (TCID_50_/ml) down to 1 TCID_50_/ml. Each viral dilution was tested in triplicate by using a modified run protocol without the automatic interpretation of results, which allowed the performance of these types of tests, assessing the cycle threshold (*C_T_*) values of each sample. Since the amplification protocol consists of 45 PCR cycles, a sample with a *C_T_* value of 40 or below was considered positive, and thus all samples with a *C_T_* of ≥40 were excluded from the trial. A tentative LoD of 10 TCID_50_/ml was assumed for both BALs and NPSs. Thus, 10 TCID_50_/ml represented the limit of the viral dilution that tested positive with both targets (reaction mix 1 and reaction mix 2) in all replicates, displaying a mean *C_T_* value below the set limit (Tables 1 and 2). The LoD was further confirmed by 20 repetitions of the previously identified tentative LoD. The NPSs displayed mean *C_T_* values of 36.746 ± 0.344 (95% confidence interval [CI], 36.402 to 37.090) for mix 1 and 37.346 ± 0.318 (95% CI, 37.029 to 37.664) for mix 2, while in the BAL matrix, the *C_T_* values were 32.948 ± 0.256 (95% CI, 32.693 to 33.205) and 34.325 ± 0.207 (95% CI, 34.118 to 34.532) for mix 1 and mix 2, respectively (Tables 1 and 2). The high sensitivity of the test was further confirmed by determining the LoD for viral genome copies. In this case, the limit of detection of the bKIT Virus Finder COVID-19 was set to 10 copies/μl for both NPS and BAL matrices (Table 3). Indeed, both reaction mixes (mix 1 and mix 2) detected up to 10 copies/μl of viral target with a mean *C_T_* of 36.640 ± 3.97 (95% CI, 32.473 to 40.806) and 36.070 ± 3.40 (95% CI, 32.501 to 39.638), respectively. In contrast, reaction mix 2 displayed a lower sensitivity at a lower dilution (2 copies/μl) of SARS-CoV-2 RNA (Table 3).

**TABLE 1:** Tentative LoD of the bKIT Virus Finder COVID-19 assay in NPS or BAL negative matrices

LoD	SARS-CoV-2 (TCID_50_/ml)	*C_T_* (mean ± SD)		
		Reaction mix 1	Reaction mix 2	RP
Tentative, in NPS	1 × 10^4^	26.89 ± 0.15	27.14 ± 0.19	25.26 ± 0.05
	1 × 10^3^	30.45 ± 0.07	30.68 ± 0.15	25.28 ± 0.03
	1 × 10^2^	33.33 ± 0.63	34.1 ± 0.16	25.2 ± 0.04
	10	38.18 ± 0.53	37.68 ± 1.01	25.09 ± 0.16
	1	N/A	N/A	25.18 ± 0.02
	
Tentative, in BAL	1 × 10^4^	27.74 ± 0.06	29.30 ± 2.02	29.91 ± 0.17
	1 × 10^3^	31.14 ± 0.47	33.33 ± 0.44	30.82 ± 0.19
	1 × 10^2^	34.13 ± 0.08	36.44 ± 0.04	31.88 ± 0.15
	10	37.29 ± 1.54	39.72 ± 0.23	33.16 ± 1.00
	1	38.52 ± 1.10	41.60 ± 0.42	32.41 ± 0.57

**TABLE 2:** CIs of LoD of the bKIT Virus Finder COVID-19 assay in NPS or BAL negative matrices

Sample	Mix	LoD (95% CI)
NPS	Mix 1	36.746 ± 0.344 (36.402–37.090)
	Mix 2	37.3465 ± 0.318 (37.029–37.664)
	RNase P	25.009 ± 0.0436 (24.965–25.053)
BAL	Mix 1	32.94894737 ± 0.256 (32.693–33.205)
	Mix 2	34.325 ± 0.207 (34.118–34.532)
	RNase P	24.5455 ± 0.152 (24.394–24.697)

**TABLE 3:** Diagnostic sensitivity evaluated by testing serial dilutions of standard viral genome copies

Sample	SARS-CoV-2 (copies/μl)	*C_T_* (mean ± SD)		
		Mix 1	Mix 2	RP
NPS	20	37.23 ± 0.91	38.61 ± 1.29	25.39 ± 0.04
	10	39.44 ± 1.58	38.47 ± 0.49	25.3 ± 0.007
	2	39.46	N/A	25.2 ± 0.03
	1	41.95 ± 1.83	N/A	25.18 ± 0.04
BAL	20	32.83 ± 0.34	32.77 ± 0.30	23.52 ± 0.46
	10	33.83 ± 0.10	33.66 ± 0.03	23.47 ± 0.17
	2	35.39	N/A	23.40 ± 0.27
	1	N/A	N/A	23.32 ± 0.25

### Specificity features of the new diagnostic assay.

The diagnostic specificity of the bKIT Virus Finder COVID-19 was also evaluated to check cross-reactivity against pathogens normally present in the upper respiratory tract. For analytical specificity evaluation, clinical specimens, culture isolates, or purified nucleic acids were added to NPS or BAL native matrix to determine cross-reactivity in three replicates. As reported in Table 4, the bKIT Virus Finder COVID-19 did not show potential false-positive RT-PCR results, especially with closely related SARS and Middle East respiratory syndrome (MERS) coronaviruses. Moreover, no interference in the detection of positive samples (*n = *8) was noticed when commonly used substances, such as oseltamivir, mupirocin, tobramycin, fluticasone, blood, oxymetazoline, and Tonimer, were added (Table 5). Likewise, the same substances failed to positively interfere with the test when added to negative samples (*n = *8). Thus, specificity and sensitivity parameters required for diagnostic purposes of the bKIT Virus Finder COVID-19 were fully confirmed.

**TABLE 4:** Specificity of the Hyris assay assessed by testing the reaction mixes with pathogens commonly present in the upper respiratory tract

Pathogen	No. of positive results/total no. (%)			
	Reaction mix 1		Reaction mix 2	
Adenovirus	0/3 (0)	0/3 (0)	0/3 (0)	0/3 (0)
Metapneumovirus	0/3 (0)	0/3 (0)	0/3 (0)	0/3 (0)
Influenza A virus	0/3 (0)	0/3 (0)	0/3 (0)	0/3 (0)
Influenza B virus	0/3 (0)	0/3 (0)	0/3 (0)	0/3 (0)
Respiratory syncytial virus	0/3 (0)	0/3 (0)	0/3 (0)	0/3 (0)
Enterovirus	0/3 (0)	0/3 (0)	0/3 (0)	0/3 (0)
Rhinovirus	0/3 (0)	0/3 (0)	0/3 (0)	0/3 (0)
Parainfluenza virus 1 to 4	0/3 (0)	0/3 (0)	0/3 (0)	0/3 (0)
Mycoplasma pneumoniae	0/3 (0)	0/3 (0)	0/3 (0)	0/3 (0)
Pneumocystis jirovecii	0/3 (0)	0/3 (0)	0/3 (0)	0/3 (0)
Chlamydia pneumoniae	0/3 (0)	0/3 (0)	0/3 (0)	0/3 (0)
Streptococcus pneumoniae	0/3 (0)	0/3 (0)	0/3 (0)	0/3 (0)
Streptococcus pyogenes	0/3 (0)	0/3 (0)	0/3 (0)	0/3 (0)
Mycobacterium tuberculosis	0/3 (0)	0/3 (0)	0/3 (0)	0/3 (0)
Pseudomonas aeruginosa	0/3 (0)	0/3 (0)	0/3 (0)	0/3 (0)
Candida albicans	0/3 (0)	0/3 (0)	0/3 (0)	0/3 (0)
Staphylococcus epidermidis	0/3 (0)	0/3 (0)	0/3 (0)	0/3 (0)
Streptococcus salivarius	0/3 (0)	0/3 (0)	0/3 (0)	0/3 (0)
Bordetella pertussis	0/3 (0)	0/3 (0)	0/3 (0)	0/3 (0)
Legionella pneumoniae	0/3 (0)	0/3 (0)	0/3 (0)	0/3 (0)
Pooled human nasal wash	0/3 (0)	0/3 (0)	0/3 (0)	0/3 (0)
Pooled SARS-CoV and MERS-CoV	0/3 (0)	0/3 (0)	0/3 (0)	0/3 (0)
Pooled OC43, NL63, 229E, and HKU-1CoVs	0/3 (0)	0/3 (0)	0/3 (0)	0/3 (0)

**TABLE 5:** Assessment of false-negative results risk

Substance	*C_T_* (mean ± SD)					
	Reaction mix 1		Reaction mix 2		RP	
	Untreated	Treated	Untreated	Treated	Untreated	Treated
Oseltamivir	25.28 ± 3.12	25.31 ± 2.58	26.14 ± 2.68	26.30 ± 2.81	25.98 ± 2.14	26.23 ± 2.27
Mupirocin	25.28 ± 3.12	25.80 ± 2.78	26.09 ± 2.72	26.80 ± 3.03	25.98 ± 2.14	26.33 ± 2.23
Tobramycin	25.22 ± 3.17	24.38 ± 1.58	26.11 ± 2.70	25.23 ± 2.20	26.15 ± 2.08	26.34 ± 2.18
Fluticasone	25.20 ± 3.18	25.40 ± 2.51	26.14 ± 2.68	26.15 ± 2.96	26.00 ± 2.13	26.84 ± 1.61
Blood	25.20 ± 3.18	25.03 ± 2.61	26.14 ± 2.68	24.98 ± 9.08	26.00 ± 2.13	26.16 ± 1.97
Oxymetazoline	25.20 ± 3.18	25.26 ± 2.50	26.14 ± 2.68	25.64 ± 9.28	26.00 ± 2.13	26.18 ± 2.19
Tonimer	25.20 ± 3.18	25.04 ± 2.63	26.14 ± 2.68	25.11 ± 9.08	26.00 ± 2.13	26.31 ± 2.40

### Diagnostic potential of the Hyris assay.

To evaluate the diagnostic performance of the bKIT Virus Finder COVID-19 assay, samples previously confirmed positive by a real-time PCR comparator assay were selected and retested with the Hyris system for SARS CoV-2. In total, 76 NPSs, of which 51 were ESwabs and 25 were in standard UTM transport medium, were collected and included in the trial for comparison. Among them, two (2/51) of the ESwab positive samples were detected as negative for SARS-CoV-2 RNA, while the RNase P internal control was successfully amplified, suggesting a partial degradation of the viral genome. Indeed, repetition of the test with a comparator assay confirmed the absence of viral target. Thus, these two samples were omitted from the final statistical evaluation. Regarding the remaining 49 specimens, a concordance of 100% (49/49) was obtained with respect to the comparator data (Tables 6 and 7). However, a discrepancy in the overall cycle threshold (*C_T_*) values was noticed for most of the ESwabs tested with the Hyris system, with a mean *C_T_* of 27.7 ± 4.52 (95% CI, 26.332 to 29.068) for mix 1 (*P < *0.0001) and 28.94 ± 5.05 (95% CI, 27.488 to 30.391) for mix 2 (*P < *0.0001), relative to the comparator *C_T_* value of 20.46 ± 3.78 (95% CI, 19.31 to 21.60) (Tables 6 and 7). In contrast, NPSs collected in the UTM medium, all (25/25) confirmed positive, provided results overlapping with the comparator values, with a mean *C_T_* of 28.74 ± 6.66 (95% CI, 25.990 to 31.489) (*P = *0.660) and 30.01 ± 6.99 (95% CI, 27.124 to 32.895) (*P = *0.287) for reaction mix 1 and 2, respectively (Tables 6 and 7). Although a direct comparison of *C_T_* values obtained with different assays is not appropriate, our data suggested that samples collected in UTM provide greater accuracy than those obtained using ESwab. Along with NPSs, a few BAL samples were included in the trial. Among them, only two (33.3%) were detected as positive, while the remaining samples were considered negative (2/6; 33.3%), since the viral genome was not recognized or was indeterminate (2/6; 33.3%) as the RNase P internal control probe was not reacting, suggesting a complete degradation of the clinical sample (Table 8). Similar results were obtained with the comparator assay. Finally, to confirm the diagnostic specificity of the new diagnostic platform, 100 NPSs confirmed SARS-CoV-2 negative were further investigated by the bKIT Virus Finder COVID-19 assay. Total accordance with the standard procedure (Seegene assay) was shown for detection of negative samples (0/100; 0%) (Tables 6 and 7).

**TABLE 6:** Diagnostic sensitivity and specificity evaluation of retrospective positive and negative samples

Parameter and sample type	No. of positive samples/total no. (%)	No. of negative samples/total no. (%)
Sensitivity		
ESwab	49/49 (100)	0/49
UTMs	25/25 (100)	0/25 (0)
BALs	2/4 (50)	2/4 (50)
Specificity		
ESwab	0/100 (0)	100/100 (100)

**TABLE 7:** Comparison of *C_T_* values of ESwabs and UTMs

bKIT mix or comparator	Mean *C_T_* ± SD (95% CI)	
	ESwabs	UTMs
Reaction mix 1	27.7 ± 4.52 (26.33–29.06)	28.74 ± 6.66 (25.99–31.48)
Reaction mix 2	28.94 ± 5.05 (27.48–30.39)	30.01 ± 6.99 (27.12–32.89)
Comparator	20.46 ± 3.78 (19.31–21.60)	27.89 ± 6.94 (25.02–30.75)

**TABLE 8:** Results evaluation based on sample reactivity with the two virus-specific (mix 1 and mix 2) probes and the internal control (RNase P) probe^*a*^

Result	Reaction mix 1	Reaction mix 2	RNase P
Positive	Pos	Pos	Pos
Negative	Neg	Neg	Pos
Inconclusive	Pos (Neg)	Neg (Pos)	Pos (Pos)
Indeterminate	Neg	Neg	Neg

a Results in parentheses indicate possible combinations of data leading to inconclusive diagnosis.

## DISCUSSION

Diagnostic performance of COVID-19 assays is the major limiting aspect of diagnostic procedures for SARS-CoV-2. Moreover, timely detection of symptomatic and asymptomatic infected subjects is crucial for stopping virus spread and for limiting its diffusion in the population, especially to vulnerable people suffering from preexisting injuries or the elderly (12). Based on these observations, it is critical that diagnostic procedures are as accurate as possible and provide results in a rapid manner to promptly isolate infected patients (8, 9, 13). Rapid tests for COVID-19 infection without underestimated diagnostic performance are urgently needed, as reported in European Directive 98/79/EC on *in vitro* diagnostic device (IVDD) guidelines (14–16). In the present study, the bKIT Virus Finder COVID-19 was evaluated, and its feasible application in routine diagnostic procedures was confirmed. Sensitivity was first evaluated based on the limit of detection (LoD) of the bKIT Virus Finder COVID-19 assay on both nasopharyngeal swabs (NPSa) and bronchoalveolar lavage fluid (BAL) samples. Our test evaluation completely satisfied sensitivity parameters. Indeed, the LoD was set with both viral N1 and N2 targets at 10 TCID_50_/ml either in NPS or in BAL. These data completely fit with the LoD reported in viral copies number. Indeed, the Hyris direct qRT-PCR method showed a viral LoD of 10 copies/μl, confirming the high sensitivity of the test. False-negative results were obtained mainly because of improper specimen collection or degradation of the viral RNA during shipping or storage. Moreover, some automatic systems for SARS CoV-2 molecular testing could be affected by the presence of high salt content in NPS transport medium, such as ESwab, providing false-negative results (7, 17). Thus, the use of NPS in UTM is strongly recommended. We noticed that the saline diluent (ESwab) for NPSs could affect the results, providing *C_T_* values higher than those obtained using UTM buffer, because of their different compositions. Therefore, we recommend the use of UTM for this system, since it better fits the gold standard parameters of the reaction. Nevertheless, in this trial, most of the clinical specimens tested using bKIT Virus Finder COVID-19, although in saline solution (ESwab), were correctly diagnosed, offering added value to this diagnostic system. Moreover, specificity (cross-reactivity) was absent, based on the analysis of several pathogens commonly present in the upper respiratory tract. Most importantly, the Hyris assay did not show reactivity with other human coronaviruses, including OC43, NL63, 229E, and HKU-1 strains, and the two SARS-CoV-2-related pandemic coronaviruses, SARS-CoV and MERS-CoV. The addition of an endogenous human RNase P gene (RP) as an internal control of the assay enabled the correct evaluation of the quality of swab sampling. Although the new kit was tested on few BAL specimens, our data demonstrated that the Hyris approach is suitable for COVID-19 diagnosis in samples other than NPSs, as long as the samples are timely processed or properly stored. In conclusion, the bKIT Virus Finder COVID-19 direct qRT-PCR by Hyris can be considered a valid and rapid diagnostic tool, providing the advantage of a less complex and unambiguous results interpretation. Moreover, the use of the Hyris platform provides the advantage of using a webpage connected to the instruments that allows remote interpretation and validation of results.

## MATERIALS AND METHODS

### Sample collection.

SARS-CoV-2-positive samples represented by nasopharyngeal swabs (NPSs) in both universal transport medium (UTM; Copan, Brescia, Italy) (*n = *25) and modified liquid Amies (ESwab; Copan) (*n = *49) were collected during the first (March to April 2020) and the second (September to October 2020) pandemic periods at the Azienda Ospedaliera Universitaria Senese Santa Maria alle Scotte Hospital in Siena, Italy. Based on comparator results with the Allplex SARS-CoV-2 assay (Seegene Inc., Seoul, Republic of Korea), we selected samples with *C_T_* values ranging from 10 to 37. One hundred previously tested negative samples were selected to evaluate the specificity of the new nucleic acid-based technology. The use of the samples was explicitly authorized for research purposes by patients at the time of their biological sampling; the study protocol (DR20003) was reviewed by the local Institutional Ethics Review Board (IERB) of the AOU Hospital of Siena (Italy), and a “favorable opinion” was expressed. The samples retrieved for the new test were anonymized and numbered in sequence. Pools of negative NPS or bronchoalveolar lavage fluid (BAL) were prepared for further validation assays. All samples were stored at −80°C until tested by the Hyris kit.

### bKIT Virus Finder COVID-19 assay.

**FIG 1:**
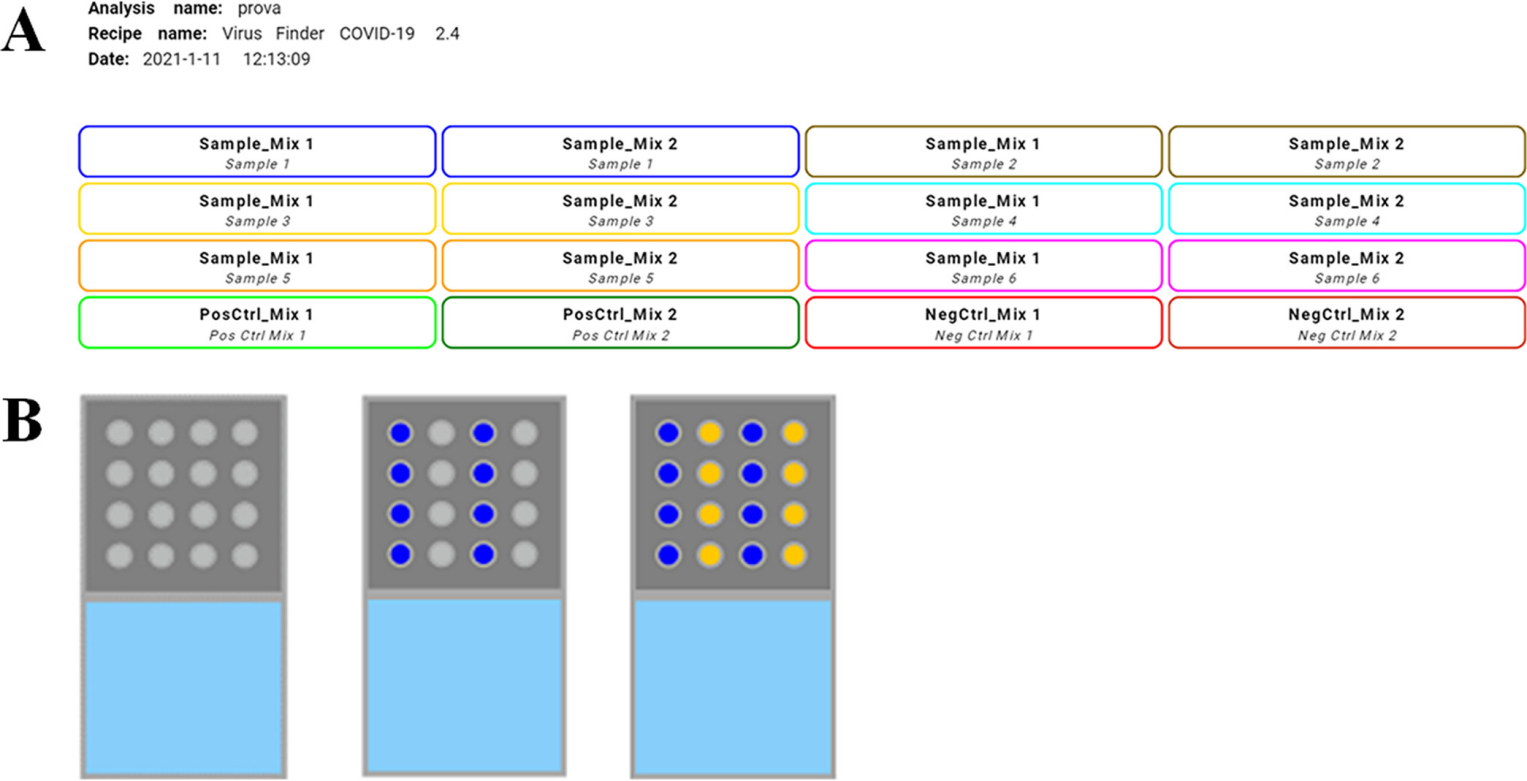
Scheme of the Hyris bCUBE cartridge. Clinical samples and positive and negative controls should be loaded on the cartridge two times, as shown by the cartridge layout (A) for testing with reaction mix 1 (blue) and reaction mix 2 (yellow) (B). Evaluation of results is automatically performed, matching all the included controls in order to avoid the generation of false-positive or false-negative data.

The bKIT Virus Finder COVID-19 amplification kit was intended for use with the bCUBE instrument (Hyris Ltd.). The diagnostic procedure was based on extraction-free real-time RT-PCR by using reaction mix 1 and mix 2 containing reverse transcriptase, DNA polymerase, RNase inhibitor, buffer with magnesium chloride, deoxynucleoside triphosphates (dNTPs), and two 6-carboxyfluorescein (FAM)-labeled fluorescent probes/primers targeting different sequences of the SARS-CoV-2 N gene (reaction mix 1 and reaction mix 2). An internal control was represented by amplification of the human RNase P gene (RP) with a HEX (6-carboxy-2,4,4,5,7,7-hexachlorofluorescein)-labeled fluorescent probe present in reaction mix 1. The testing procedure required the loading into two reaction wells of a bCUBE 16-well cartridge of each sample (5 μl) in both reaction mixes (15 μl). Positive and negative controls were also loaded in both reaction mixes. The cartridge was sealed with adhesive film in order to avoid liquid evaporation and cross-contamination among samples and the positive control. Samples and controls were loaded on the cartridge in accordance with the manufacturer’s instructions (Fig. 1). The prepared cartridge was then slotted into the bCUBE, and the experiment was run by standard protocol as defined by the manufacturer. Results were obtained within 2 h and expressed as described in Table 8.

### Pathogenic microorganisms and cross-reactivity assay.

Mycobacterium tuberculosis DNA, purified from a clinical sample, was kindly provided by F. Santoro (Laboratory of Molecular Microbiology and Biotechnologies, S. Maria delle Scotte University Hospital, Siena). Mycoplasma pneumoniae, Pneumocystis jirovecii, Chlamydia pneumoniae genomic DNA, and HKU-1 human coronavirus (CoV) genomic RNA were purified by the EZ1 system (Qiagen, Milan, Italy) from clinical samples collected at the Microbiology and Virology Unit of the S. Maria delle Scotte University Hospital in Siena. Whole genomic RNA for OC43, NL63, and 229E CoV strains and highly pathogenic SARS-CoV and MERS-CoV were purchased from the European Virus Archive (EVAg; reference number SKU 011N-03). Preliminary quality tests for purified nucleic acids were done by conventional RT-PCR or PCR. The minimum amount of DNA/RNA giving a detectable signal (*C_T_* ≤ 35) was selected for further analysis with the bKIT Virus Finder COVID-19. The selected volume of nucleic acids was directly added to the NPS and BAL negative matrices for cross-reactivity screening. Pools of SARS/MERS-CoV or OC43, NL63, 229E, and HKU-1 CoV extracts were tested. In the event of a positive result, each pathogen was individually tested to identify which one was responsible for cross-reactivity. Where applicable, virus cultures were directly added to negative matrices (NPS or BAL) to a minimum concentration of 1 × 10^4^ TCID_50_/ml. Bacteria were collected from freshly streaked plates and diluted in sterile isotonic solution to a final concentration of 1 × 10^8^ CFU/ml by using the McFarland method. After predilution in sterile isotonic solution, 1 × 10^6^ CFU/ml were inoculated in both negative matrices.

### Limit of detection.

SARS-CoV-2 was isolated at the University Hospital Santa Maria alle Scotte of Siena (Tuscany, Italy) from a nasopharyngeal swab seeded on Vero E6 cells (ATCC CRL-1586). Virus isolate Siena-1/2020 was fully sequenced and annotated in GenBank (accession no. MT531537). Live viral stock was used to test the sensitivity of the bKIT Virus Finder COVID-19 assay by limit of detection (LoD). Sample preparation was performed in a biosafety level 3 (BSL3) laboratory by serial dilution of the virus in NPS or BAL negative matrix starting from 1 × 10^5^ to 10 TCID_50_/ml. Three repeats for each dilution were tested in the trial, and the tentative LoD of the assay was determined as the lowest detectable virus titer at which >95% of replicates tested positive. The LoD of the assay was confirmed by 20 repeats of the tentative LoD. The significance of the results was evaluated by the 95% confidence interval. The limit of detection (LoD) was further evaluated on the SARS-CoV-2 RNA standard (Exact Diagnostics, TX, USA). The reference RNA standard was serially diluted (ranging from 20 to 1 copy/μl) in negative NPS or BAL matrices before bKIT Virus Finder COVID-19 test assessment. Results are presented as the mean *C_T_* value ± standard deviation (SD) of two independent trials.

### Interference assay.

Eight retrospective known positive specimens and 8 known negative specimens were tested again with and without the addition of potential interfering substances. Mupirocin, tobramycin, and oseltamivir were purchased from Sigma-Aldrich (Milan, Italy). Stock solutions were prepared according to the manufacturer’s instructions and then diluted to 6.6 mg/ml, 4 μg/ml, and 3.3 mg/ml, respectively, in the matrices. Commercial pharmaceutical formulations containing oxymetazoline (Vicks Sinex), fluticasone (Flixonase), and isotonic saline solution (Tonimer Lab) have been used as a source of the corresponding interfering substances. Each pharmaceutical product was diluted in the positive or negative matrices to 15% (vol/vol), 5% (vol/vol), and 5% (vol/vol) final concentrations. Whole blood containing anticoagulant was drawn from healthy donors and diluted in sterile physiological saline solution to obtain a 20% (vol/vol) stock solution and then to 2% (vol/vol) in the matrices. Each positive sample added with the potentially interfering substance was tested in triplicate, while each negative sample added with the interfering substance was tested in a single replicate. Results are presented as the mean ± standard deviation (SD) of *C_T_* values.
